# PGC1α/SIRT3-Mediated Oxidative Stress and Downregulation of NLRP3 Expression by Sodium Thiosulfate Pre- and Post-Treatment Accelerates Recovery from Ischemia–Reperfusion-Induced Acute Kidney Injury

**DOI:** 10.3390/antiox15101256

**Published:** 2026-10-01

**Authors:** George J. Dugbartey, Liam McFarlane, Talal Shamma, Jifu Jiang, Sally Major, Aaron Haig, Alp Sener

**Affiliations:** 1 Department of Surgery, Division of Urology, London Health Sciences Center, Western University, London, ON N6A 5A5, Canada; gdugbart@uwo.ca; 2 Matthew Mailing Center for Translational Transplant Studies, London Health Sciences Center, Western University, London, ON N6A 5A5, Canada; 3 Department of Physiology & Pharmacology, Western University, London, ON N6A 5C1, Canada; 4 Department of Physiology and Pharmacology, Accra College of Medicine, East Legon, Accra P.O. Box CT 9828, Ghana; 5 Department of Microbiology & Immunology, Schulich School of Medicine & Dentistry, Western University, London, ON N6A 5C1, Canada; 6 Department of Pathology, Schulich School of Medicine & Dentistry, Western University, London, ON N6A 5C1, Canada

**Keywords:** acute kidney injury (AKI), ischemia–reperfusion injury (IRI), sodium thiosulfate (STS), sirtuin 3 (SIRT3), peroxisome proliferator-activated receptor-gamma coactivator 1-alpha (PGC1α)

## Abstract

**Background:** Renal ischemia–reperfusion injury (IRI) is the leading cause of acute kidney injury (AKI). It is associated with reduced blood flow as encountered in vascular surgery, including aortic aneurysm repair and kidney transplantation. In this study, we investigated whether pre- and/or post-administration of sodium thiosulfate (STS), an FDA-approved hydrogen sulfide donor drug, attenuated IRI-induced AKI in rats. **Methods:** AKI was induced in male rats by clamping both renal arteries for 60 min. At 30 min before clamping, and 30 min after reperfusion, STS was administered at a weigh-based dose to achieve a circulating concentration of 150 μM, after which the rats were kept in metabolic cages for urine and blood collection on postoperative days (POD) 3, 5, and 7. Rats were sacrificed on POD3, POD5, and POD7, and kidneys were harvested for analysis. Sham-operated rats received no treatment. **Results:** AKI was evidenced by markedly higher acute tubular necrosis score, and renal protein and gene expression of damage markers (KIM-1 and NGAL), as well as markers of oxidative stress (MDA, GSH, and SOD), inflammation (MPO, IL-6), and apoptosis (TUNEL). While significant incremental improvement was observed in the expression of these markers in the pre-treatment only and post-treatment only groups over the POD3–7 observation period compared to untreated control group (*p* < 0.05), superior renal protection was observed in the pre + post-treatment group (*p* < 0.01). Functionally, serum creatinine levels were significantly higher over the POD3–7 observation period in the untreated group relative to pre-treatment and post-treatment groups (*p* < 0.05), and markedly lower in the pre + post-treatment group (*p* < 0.001), which positively correlated with urine output. Urine osmolality was statistically similar to sham-operated rats on POD5 and 7, while all other groups showed decreased urine osmolality on POD3 and 5 (*p* < 0.05). Mechanistically, the protective effect of STS correlated with upregulation of PGC1α and SIRT3 protein and gene expression and downregulation of NLRP3 protein and mRNA expression in the pre + post-treatment group compared to other groups (*p* < 0.05). **Conclusions:** The present study reports for the first time the protective effect of STS against IRI-induced AKI, and indicates PGC1α, SIRT3, and NLRP3 as molecular targets of STS.

## 1. Introduction

Acute kidney injury (AKI), formerly known as acute renal failure, is a common clinical condition with a sudden decline in kidney function characterized by a rapid increase in the levels of nitrogenous waste compounds such as serum creatinine and blood urea nitrogen, reduction in glomerular filtration rate, and/or decrease in urine output [1,2,3]. It is associated with high mortality rate, longer hospital stays, significant financial burden, and long-term adverse outcomes such as progression to chronic kidney disease, end-stage renal disease, and cardiovascular morbidity, which negatively impact quality of life and may require personalized and precision-oriented management [2,3]. AKI is a major global public health concern, affecting about 13.3 million patients annually, with over 50% in intensive care units and about 1.7 million deaths every year [3,4]. Among several factors contributing to the high incidence of AKI, renal ischemia–reperfusion injury (IRI) is universally recognized as the leading cause of AKI as encountered in many clinical conditions involving poor renal perfusion, such as in major surgeries (e.g., kidney transplantation, cardiac surgery, and vascular surgery) [5,6,7,8]. Renal IRI is a pathological condition arising from sudden temporal cessation or reduction of blood supply to the kidney followed by its restoration. Considering that the kidneys are among the metabolically active organs, receiving about 25% of cardiac output, and with the medulla receiving only about 10% of the renal perfusion [9,10], the renal medulla, together with the unique anatomy of its vascular bed, represents a natural hypoxic environment that is vulnerable to IRI-induced AKI.

While intrarenal oxidative stress, inflammation, and tubular cell death have been implicated in IRI-induced AKI [11,12], the molecular mechanism is complex and remains poorly understood. Also, effective pharmacotherapy and therapeutic strategies to prevent and accelerate recovery from IRI-induced AKI are lacking, despite the high incidence and severity of the condition and advances in diagnosis and patient management. These challenges suggest the need for a comprehensive understanding of the molecular mechanisms of IRI-induced AKI in order to identify effective therapeutic strategies. Sodium thiosulfate (STS), a clinical drug originally designed as an antidote for acute cyanide poisoning [13,14], is now being repositioned to ameliorate non-renal IRI in preclinical models [15,16,17,18]. However, similar studies addressing renal IRI are lacking. Moreover, current treatment for AKI is mainly supportive in nature without therapeutic modalities, and preventive treatment protocols also face major challenges, as the serum creatinine level rises only after significant loss of kidney function [19,20]. We recently observed renoprotective effects of STS in rat models of kidney transplantation [7,21,22]. Using a rat model of IRI-induced AKI, we aim to determine the preventive and therapeutic impacts of STS and its underlying molecular mechanisms of action.

## 2. Materials and Methods

### 2.1. Ethical Statement and Experimental Animals

This study was conducted in accordance with relevant national and international guidelines and was approved by the University of Western Ontario Animal Care Committee (Protocol #: 2022-093) on 12 January 2022, adhering to protocol and maintaining good laboratory practice.

One hundred and five (105) male Sprague-Dawley rats weighing 300–400 g were purchased at 10-weeks-old from Charles River Laboratories, Quebec, Canada. The rats were housed under normal light–dark conditions (12:12 h) at 20–25 °C ambient temperature and a humidity range of 40–60% and were fed ad libitum with standard rat chow and tap water.

### 2.2. Animal Grouping and Creation of Renal IRI-Induced AKI

**Figure 1:**
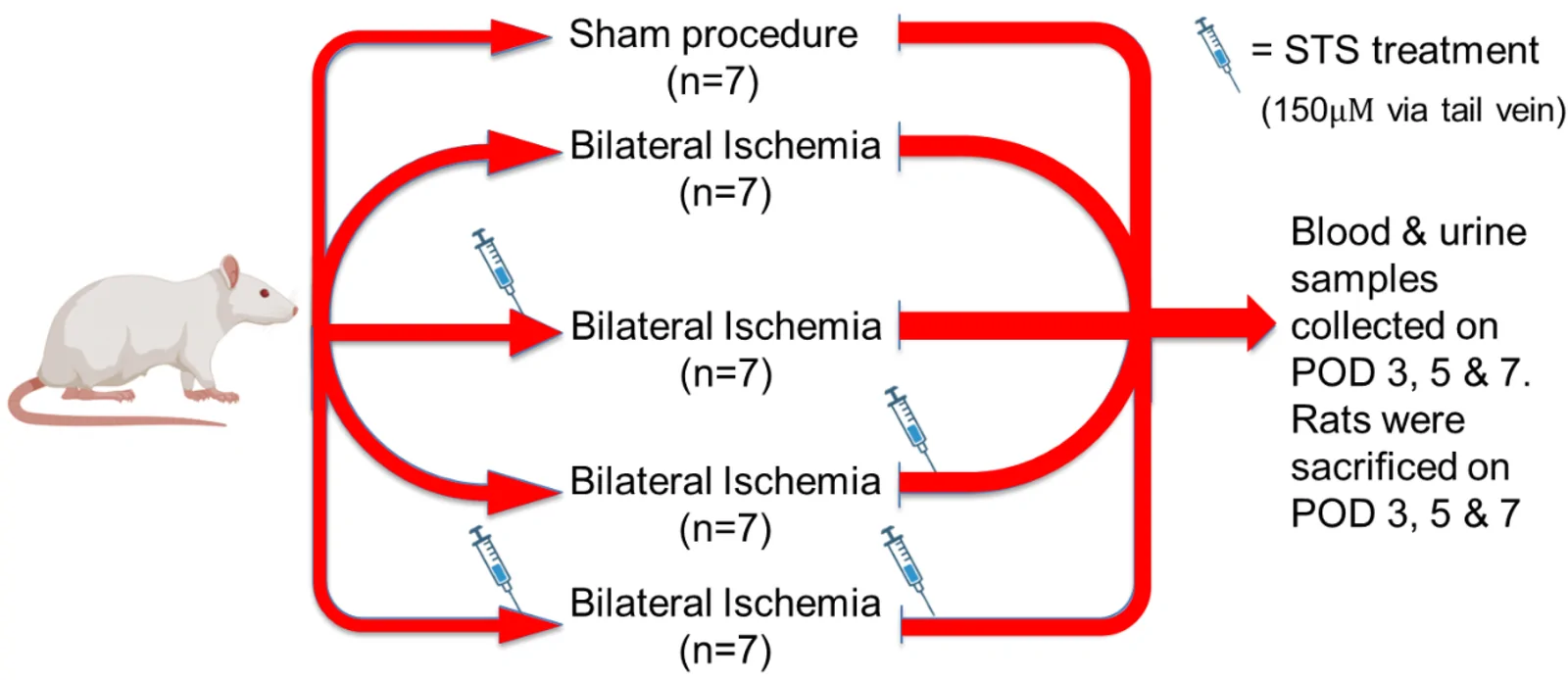
Experimental model of IRI-induced AKI. Rats were randomly assigned to 5 groups, and STS was administered before and/or after induction of IRI.

Rats were first randomized into sham, untreated control, and treatment groups. Intravenous injections of 0.2 mL saline or 150 µM STS were administered via the tail vein 30 min before induction of renal ischemia and 30 min after the restoration of renal perfusion. Treatment groups included STS administration before surgery and saline injection post-operatively (pre-treatment group, n = 7), saline injection before surgery and STS administration post-operatively (post-treatment group, n = 7), STS administration before and after surgery (pre- and post-treatment group, n = 7). The control groups were sham (only laparotomy to establish baseline; n = 7) and untreated control (only saline injection; n = 7). Anesthesia was induced using ketamine (100 mg/mL) and xylazine (20 mg/mL) and maintained using isoflurane throughout the surgery. Buprenorphine (0.1 mg/kg) was administered subcutaneously at the time of anesthesia induction. After laparotomy, bilateral occlusion of the renal pedicles was performed using micro-vessel clips for 60 min to induce ischemic damage in the kidney. Following clip removal, the abdominal incision was closed, and renal function was monitored over the following 7 days, taking blood and urine samples on post-operative days (PODs) 3, 5, and 7. Rats were sacrificed on POD3, 5, and 7 and kidneys were harvested for analysis. All surgeries were performed by the same microsurgeon who was blinded to all experimental groups. Animal welfare was assessed twice daily throughout the experiment according to the standard operating procedure set by Animal Care and Veterinarian Services. The experimental model is depicted graphically in Figure 1. Rats were used for this model because they are known to recover effectively from AKI due to robust tubular cell proliferation, rapid adaptive tissue repair, and a strong antioxidant defense response [23,24,25,26].

### 2.3. Preparation of Serum and Urine Samples for Biochemical Analysis

Following AKI-inducing surgical procedure, rats were placed in metabolic cages on PODs 2, 4, and 6 for 24 h for urine collection the next day. The volume of water intake and urine output were recorded for each day spent in the metabolic cage. To prepare serum samples, about 200 µL of blood was collected from the saphenous vein under isoflurane anesthesia on PODs 3, 5, and 7. The blood was collected in an Eppendorf tube and allowed to clot, after which the clotted blood was spun at 2000× *g* for 15 min at 4 °C. The supernatant (serum) was collected, and serum creatinine was measured using an IDEXX Catalyst One Chemistry Analyzer machine (IDEXX, Westbrook, ME, USA). Urine osmolality was determined by freezing-point osmometry using a 3320 Osmometer machine (Advanced Instruments, Norwood, MA, USA).

### 2.4. Determination of Renal Antioxidant Status

#### 2.4.1. Measurement of Reactive Oxygen Species in Kidney Tissue

Renal production of reaction oxygen species (ROS) was determined by measuring the level of malondialdehyde (MDA) spectrophotometrically after 50 mg of kidney tissue was homogenized with PBS containing butylated hydroxytoluene. Results were expressed as nanomoles of MDA per milligram of kidney tissue, according to a previously described method [27].

#### 2.4.2. Measurement of Renal Antioxidants

Using a GSH-Glo™ kit from Promega (Madison, WI, USA), glutathione (GSH) content in kidney tissue was measured as previously described [28]. In a nutshell, about 50 mg of kidney tissue samples was homogenized in 1 mL of cold KCl (0.5%) and sonicated for 60 s followed by centrifugation at 3000 rpm for 10 min at 4 °C temperature. The supernatant was collected and used together with GSH standard provided by the manufacturer, pipetted in a SpectraMax 2 plate reader (Molecular Devices, San Jose, CA, USA). Next, the amount of GSH in kidney tissue was quantified by chemiluminescence following the manufacturer’s instructions. Superoxide dismutase is an antioxidant enzyme in renal tissue; its activity was measured using a test kit (Nanjing Kaiji Bio, Nanjing, China) and 50 mg of kidney tissue, in accordance with the manufacturer’s manual and as previously described [29].

### 2.5. Histopathology and Immunohistochemical Staining

Formalin-fixed kidneys were embedded in paraffin and sectioned at 4 µm thick from the renal cortex and in similar plane. The paraffin-embedded kidney sections were dewaxed and stained with hematoxylin and eosin (H&E). The stained kidney sections were examined blindly by renal a pathologist, and acute tubular necrosis (ATN) was quantified using the following scoring scale: 1 ≤ 11%, 2 = 11–24%, 3 = 25–45%, 4 = 46–75%, 5 ≥ 75% [6].

### 2.6. Reverse Transcription Quantitative Polymerase Chain Reaction

Total RNA was isolated from kidney tissues obtained at PODs 3, 5, and 7 using an RNeasy^®^ Mini Kit (Qiagen, Toronto, ON, Canada) and reverse transcribed into cDNA using a OneScript^®^ Plus cDNA synthesis kit (ABM, Richmond, BC, Canada) with oligo(dt) primers according to the manufacturer’s protocol. Isolated RNA concentrations were assessed using a UV5 Nano spectrophotometer (Mettler Toledo, Columbus, OH, USA). qPCR reaction mixtures included BlastTaqTM 2X qPCR MasterMix (ABM, Richmond, BC, Canada) and were set up according to the manufacturer’s protocol. RT-qPCR was performed using a QuantStudio™ 3 Real-Time PCR System (Applied Biosystems, Waltham, MA, USA). All genes of interest were normalized to GAPDH (housekeeping gene), and primer sequences were designed using Primer–BLAST software version 2.5.0 (NCBI), as shown in Table 1.

### 2.7. Statistical Analysis

The data was analyzed using one-way analysis of variance (ANOVA) and a subsequent Tukey’s post hoc test. These tests were performed using GraphPad Prism 9 (version 9.5.1; San Diego, CA, USA). Statistical significance between groups was accepted when *p* < 0.05. Values are presented as mean ± standard error of the mean (SEM). A statistical power of 0.80 was computed using the formula: power = 1 − β, where β is Type II error probability based on acceptable risk of missing a real effect.

## 3. Results

### 3.1. Pre- and Post-Treatment with STS Preserved Renal Tubular Structure Against IRI-Induced AKI

**Figure 2:**
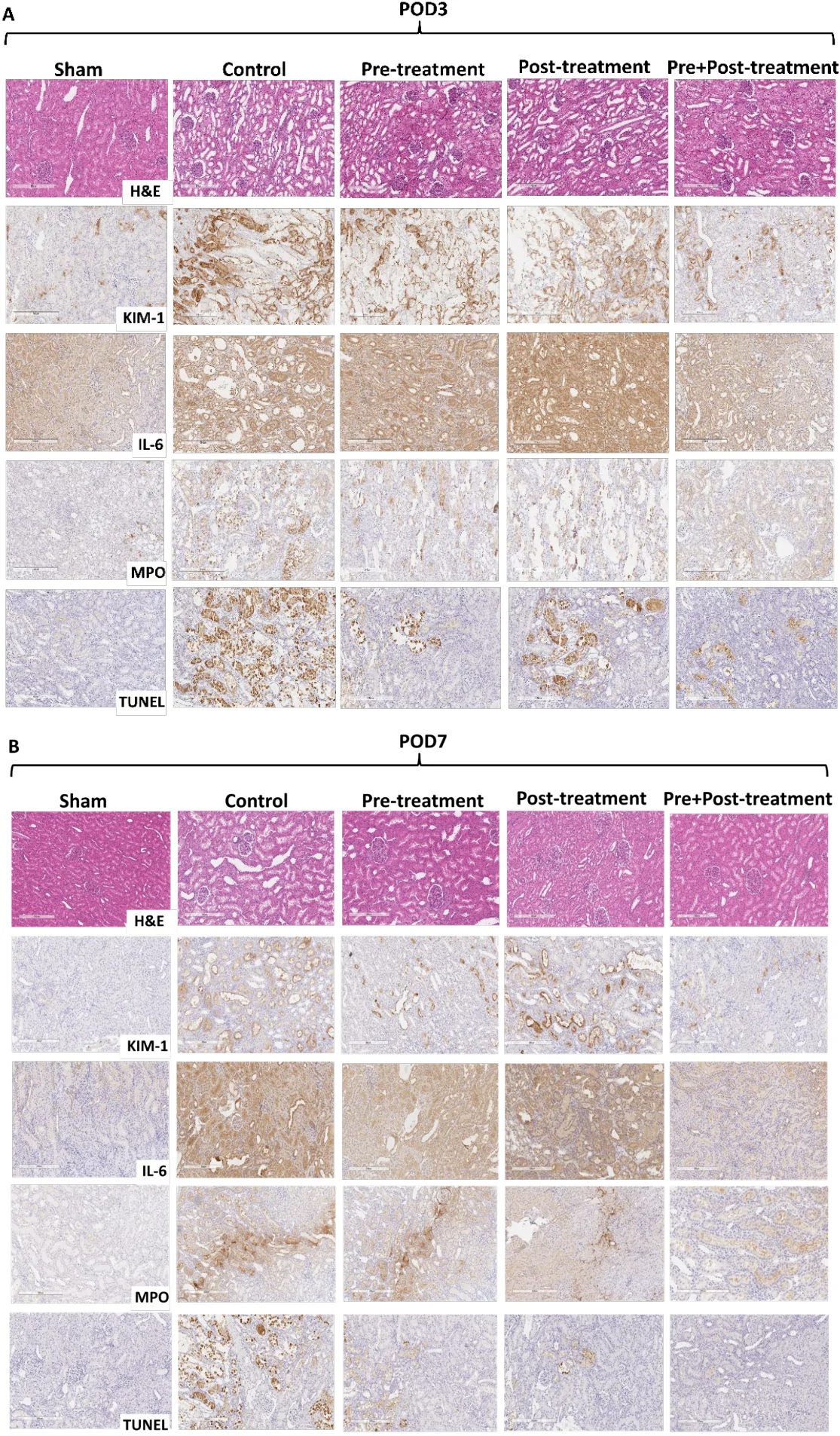
Representative images of immunohistochemical staining of kidney tissues on (**A**) post-operative day 3 (POD3) and (**B**) post-operative day 7 (POD7), showing hematoxylin and eosin staining (H&E), kidney injury molecule-1 (KIM-1), interleukin-6 (IL-6), myeloperoxidase (MPO), and terminal deoxynucleotidyl transferase dUTP nick-end labeling (TUNEL) staining. H&E staining shows renal morphology, KIM-1 indicates renal tubular injury, IL-6 and MPO denote inflammation, and TUNEL indicates apoptosis.

**Figure 3:**
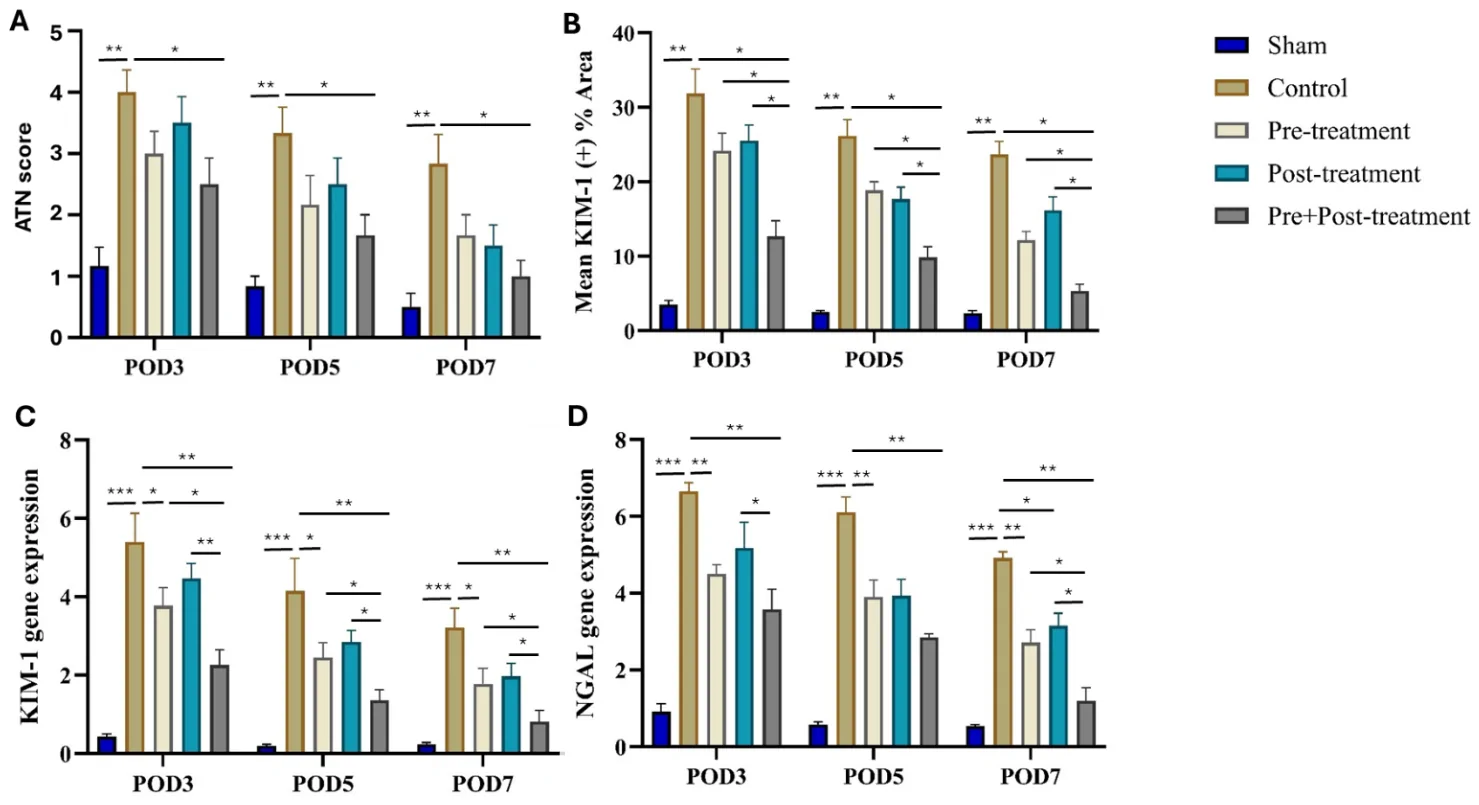
Quantification of immunohistochemical staining (**A**,**B**) and renal mRNA expression of KIM-1 and NGAL (**C**,**D**). While pre-treatment and post-treatment groups showed incremental improvement in renal injury from POD3–7 compared to the control group, the pre + post-treatment group showed superior renal protection compared to all experimental groups. n = 7 in each group. Values are presented as mean ± SEM. */**/*** *p* < 0.05/0.01/0.001.

To determine the effect of pre- and post-treatment with STS on renal architecture during IRI-induced AKI, we assessed renal histopathological changes and gene expression of markers of renal injury on post-operative days (PODs) 3, 5, and 7. Compared to the sham group, histopathological examination revealed significant renal injury in the control group from POD3–7, as assessed by H&E and KIM-1 stains (Figure 2A,B and Figure 3A,B; *p* < 0.01) and renal mRNA expressions of KIM-1 and NGAL genes (Figure 3C,D; *p* < 0.001). While incremental improvements in renal injury were observed in STS pre-treatment and post-treatment rats compared to control rats over the 7-day observation period after surgery (Figure 2A,B and Figure 3A–D), superior renal protection was observed in the STS pre + post-treatment group compared to the control, STS pre-treatment, and post-treatment groups of rats (Figure 2A,B and Figure 3A–D; *p* < 0.05); these results were comparable to the sham group (Figure 2A,B and Figure 3A–D). Interestingly, no significant difference in these renal injury markers was observed between the STS pre-treatment and post-treatment groups (Figure 2A,B and Figure 3A–D). Taken together, pre + post-treatment with STS protected renal architecture against IRI-induced AKI.

### 3.2. Pre- and Post-Treatment with STS Reduced Inflammation and Apoptosis Against IRI-Induced AKI

**Figure 4:**
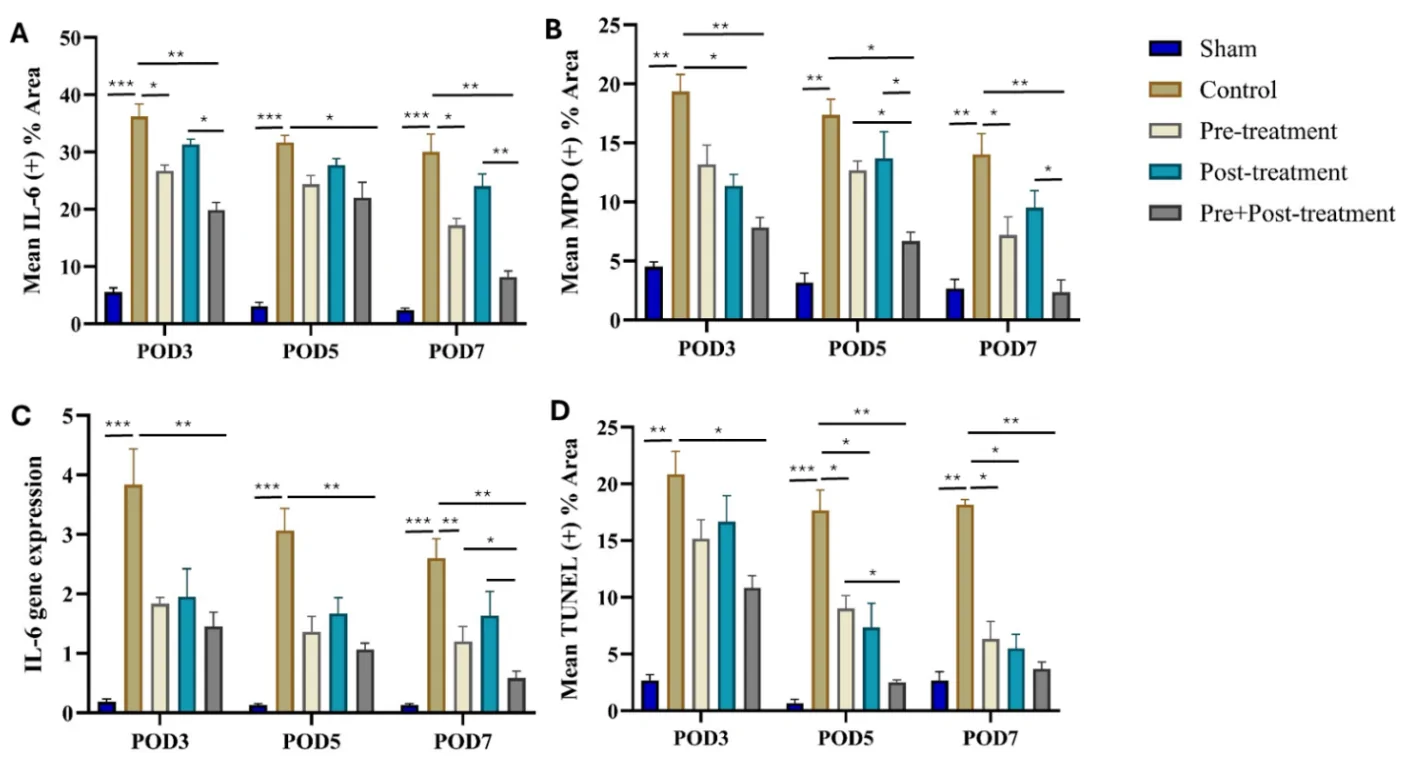
Quantification of immunohistochemical staining (**A**,**B**,**D**) and renal mRNA expression of IL-6 (**C**), showing reduced renal inflammation and apoptosis by STS in pre-treatment and post-treatment groups from POD3–7 compared to the control group. The pre + post-treatment group showed superior anti-inflammatory and anti-apoptotic effects compared to all experimental groups. n = 7 in each group. Values are presented as mean ± SEM. */**/*** *p* < 0.05/0.01/0.001.

To identify whether inflammation and apoptosis contributed to IRI-induced AKI, and to evaluate the impact of pre- and post-treatment with STS on these parameters, we performed immunohistochemical staining of the kidney with interleukin-6 (IL-6), myeloperoxidase (MPO), and TUNEL, and further assessed renal IL-6 gene expression. Immunohistochemical staining showed marked renal expression of IL-6 and MPO and apoptosis in control rats relative to sham rats from POD3–7 (Figure 2A,B and Figure 4A,B,D; *p* < 0.01), which corresponded with IL-6 gene expression (Figure 4C; *p* < 0.001). Kidneys in the STS pre-treatment and post-treatment groups showed significant downregulation of these injury markers in comparison with control kidneys over the 7-day post-operative period (Figure 2A,B and Figure 4A–D; *p* < 0.05), although there was no substantial difference in renal expression of these markers between the STS pre-treatment and post-treatment groups of rats (Figure 2A,B and Figure 4A–D; *p* > 0.05). Remarkably, rats in the STS pre + post-treatment group displayed markedly downregulated renal expression of these damage markers compared to all experimental groups (Figure 2A,B and Figure 4A–D; *p* < 0.05), which was not significantly different from the sham group by POD7 (Figure 2A,B and Figure 4A–D; *p* > 0.05). In summary, pre + post-treatment with STS reduced inflammation and apoptosis in IRI-induced AKI.

### 3.3. Pre- and Post-Treatment with STS Restored Renal Function and Preserved Renal Antioxidant Status Against IRI-Induced AKI

**Figure 5:**
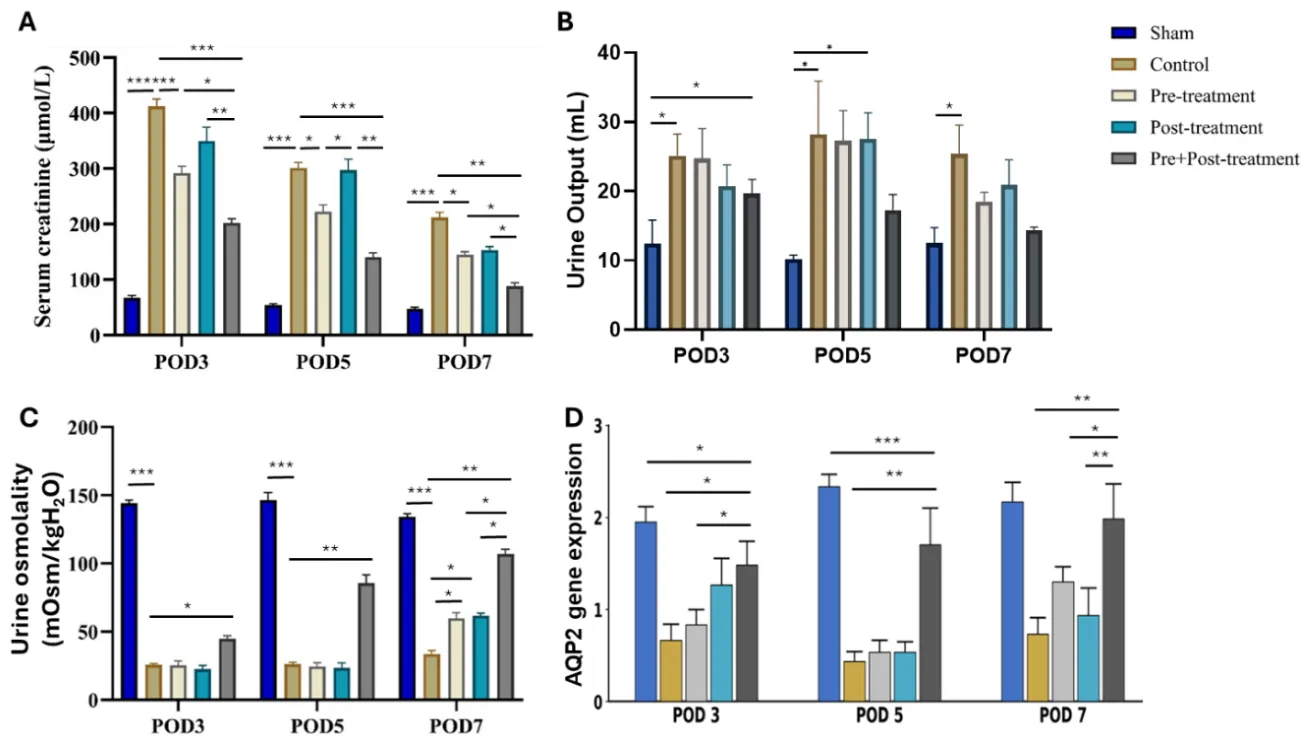
STS restored renal function against IRI-induced AKI. (**A**) Serum creatinine, (**B**) urine output, (**C**) urine osmolality, and (**D**) aquaporin 2 (AQP2) gene expression. STS pre-treatment and post-treatment reversed renal dysfunction against IRI-induced AKI by POD7. The pre + post-treatment group showed superior renoprotective effect compared to all experimental groups. n = 7 in each group. Values are presented as mean ± SEM. */**/*** *p* < 0.05/0.01/0.001.

To assess renal function during IRI-induced AKI from POD3–7, we measured serum creatinine, urine production, urine osmolality, and renal aquaporin 2 (AQP2) gene expression. IRI-induced AKI resulted in significantly higher serum creatinine level and urine output in the control group than in the sham group (Figure 5A,B; *p* < 0.001), which correlated negatively with urine osmolality and renal AQP2 expression (Figure 5C,D; *p* < 0.001). While the STS pre-treatment and post-treatment groups showed gradual reversal of the pathological levels of these renal function markers compared to control rats over the 7-day post-operative period (Figure 5A–D; *p* < 0.05), there was no statistical difference between the STS pre-treatment and Ppst-treatment groups. However, rats in the STS pre + post-treatment group showed the most remarkable reversal and restoration of renal function in comparison with all experimental groups (Figure 5A–D; *p* < 0.05), bringing the levels of these parameters in the STS pre + post-treatment group to sham levels (Figure 5A–D; *p* > 0.05).

**Figure 6:**
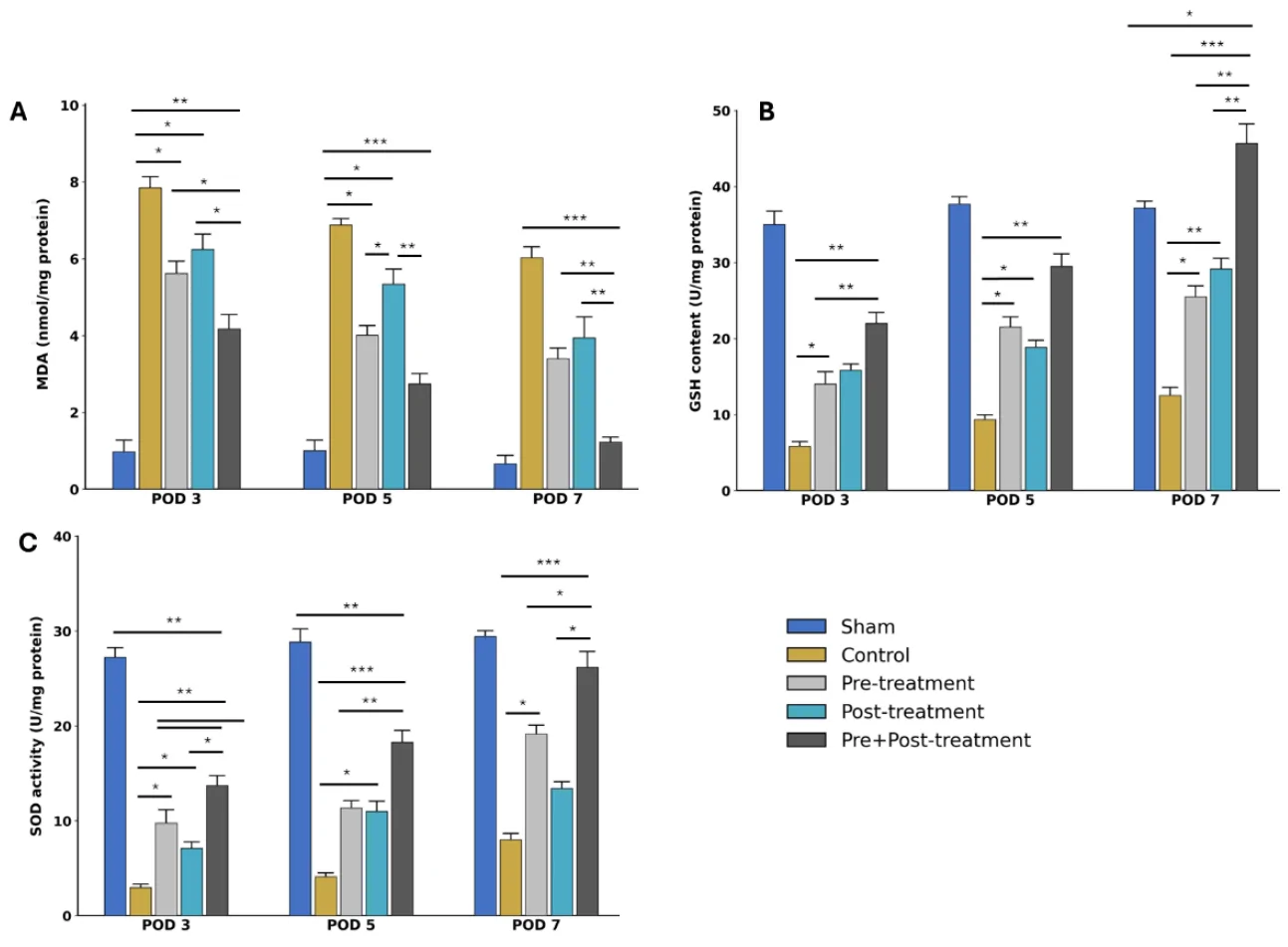
STS preserved renal antioxidant status during IRI-induced AKI. (**A**) MDA, (**B**) GSH, and (**C**) SOD. STS pre-treatment and post-treatment improved and preserved renal antioxidant status during IRI-induced AKI. The pre + post-treatment group showed superior antioxidant effect compared to all experimental groups. n = 7 in each group. Values are presented as mean ± SEM. */**/*** *p* < 0.05/0.01/0.001.

We also measured renal antioxidant status from POD3–7 by measuring renal malondialdehyde (MDA), glutathione (GSH) content, and superoxide dismutase (SOD) activity. Similar to renal function, IRI-induced AKI was associated with significantly elevated renal MDA level, along with markedly reduced renal GSH content and SOD activity in control rats compared to sham rats from POD3–7 (Figure 6A–C; *p* < 0.001). Rats in the STS pre-treatment and post-treatment groups showed substantial decreases in renal MDA levels and marked increases in renal GSH content and SOD activity by POD7, with further improvement in pre + post-treatment group to a level comparable to the sham group (Figure 6A–C). Interestingly, renal GSH content in pre + post-treatment rats was significantly higher than in sham rats (Figure 6B; *p* < 0.06). Collectively, pre + post-treatment with STS restored renal function and improved renal antioxidant status against IRI-induced AKI.

### 3.4. Renal Protection by STS Against IRI-Induced AKI Correlates with PGC1α/SIRT3 Upregulation and NLRP3 Downregulation

**Figure 7:**
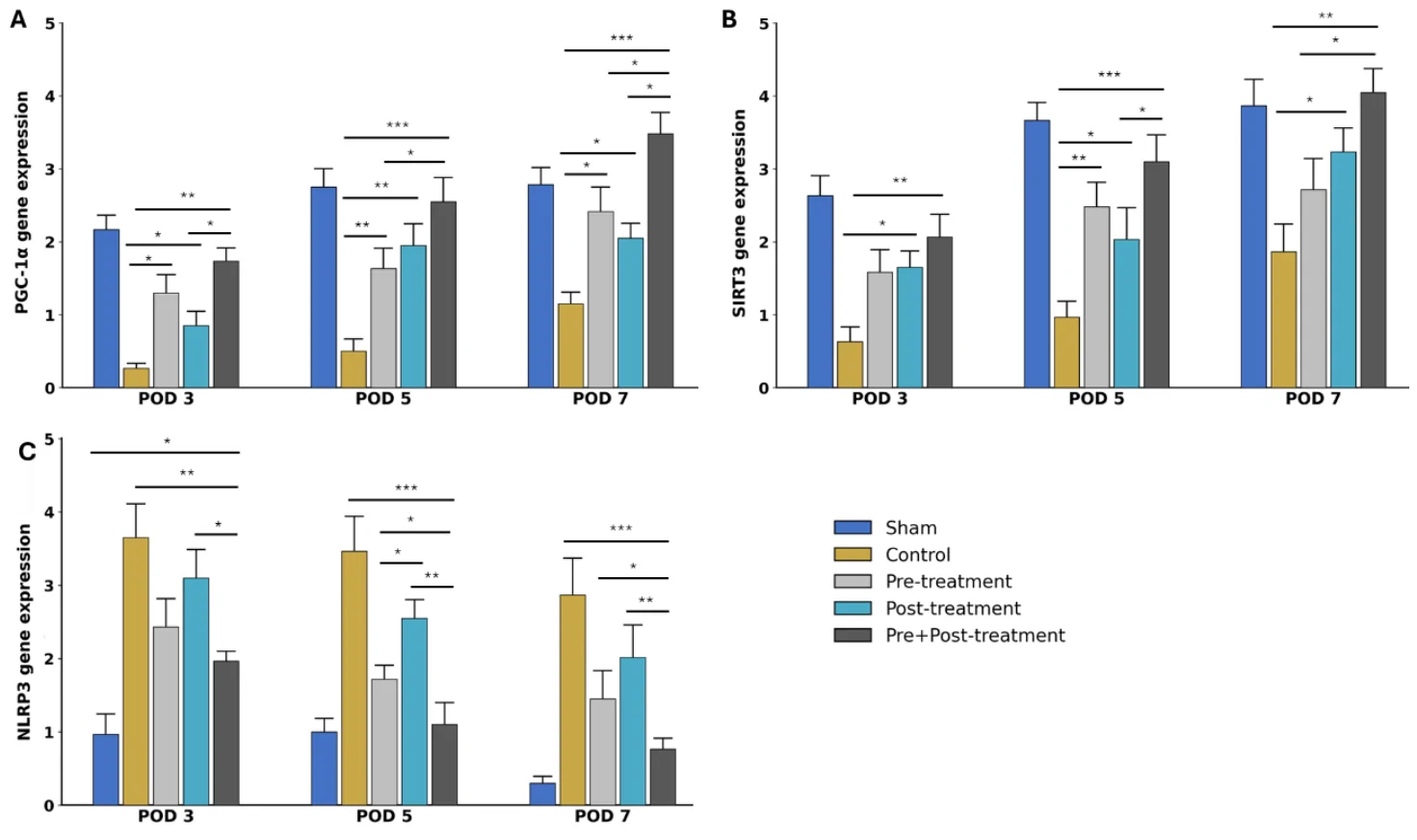
Mechanism of STS protection against IRI-induced AKI. (**A**) PGC-1α, (**B**) SIRT3, and (**C**) NLRP3. STS upregulated renal PGC1α and SIRT3 gene expression while downregulating renal NLRP3 gene expression. Thus, renal protection by STS against IRI-induced AKI is via activation of PGC1α/SIRT3-mediated NLRP3 inhibition, exemplified in the STS pre + post-treatment group. n = 7 in each group. Values are presented as mean ± SEM. */**/*** *p* < 0.05/0.01/0.001.

To investigate the mechanism underlying the observed renal protection by STS against IRI-induced AKI, we measured renal mRNA expression of PGC1α (peroxisome proliferator-activated receptor gamma coactivator 1-alpha), SIRT3 (sirtuin 3), and NLRP3 (NOD-like receptor protein 3). In comparison with the sham group, renal PGC1α and SIRT3 genes were significantly downregulated from POD3–7 in untreated control rats, which negatively correlated with renal NLRP3 gene expression (Figure 7A–C; *p* < 0.001). However, renal PGC1α and SIRT3 genes were markedly upregulated in STS pre-treatment and post-treatment rats along with significant downregulation of the renal NLRP3 gene relative to the control group (Figure 7A–C; *p* < 0.05). Interestingly, while no difference in renal PGC1α and SIRT3 gene expression was observed in the STS pre-treatment and post-treatment groups over the 7-day post-operative period (Figure 7A,B; *p* > 0.05), NLRP3 gene expression was significantly higher in the kidneys of STS post-treatment rats than in the STS pre-treatment group on POD5 (Figure 7C; *p* < 0.05). Noticeably, renal PGC1α and SIRT3 genes were substantially upregulated in STS pre + post-treatment rats compared to the control, STS pre-treatment, and post-treatment groups of rats from POD3–7 (Figure 7A,B; *p* < 0.05), with a corresponding downregulation of NLRP3 gene (Figure 7C; *p* < 0.05). Altogether, renal protection by STS against IRI-induced AKI correlated with upregulation of PGC1α and SIRT3 and downregulation of NLRP3 genes and proteins, which was exemplified in the STS pre + post-treatment group.

## 4. Discussion

Renal IRI is a major contributor to AKI. In the present study, we investigated the pharmacological effect of STS and its underlying molecular mechanisms of action in a rat model of IRI-induced AKI. The present study showed that IRI-induced AKI was associated with tubular injury, inflammation, and apoptosis as observed in immunohistochemical stains (H&E, KIM-1, IL-6, MPO and TUNEL) from POD3–7. Functionally, IRI-induced AKI in the current study was characterized by elevated serum creatinine and urine production along with reduced urine osmolality and downregulated renal aquaporin-2 (AQP2) gene expression, indicating impaired renal function. Interestingly, while separate pre- and post-treatments with STS reduced the development of this pathology, STS pre + post-treatment preserved renal architecture and function, as evidenced by significantly lower acute tubular necrosis score, reduced expression of KIM-1, markers of inflammation and apoptosis, serum creatinine, and increased urine osmolality and AQP2 expression. These results were comparable to those from kidneys from the sham-operated group without IRI-induced AKI. In addition, STS pre + post-treatment prevented oxidative stress and maintained renal antioxidant status throughout the 7-day post-operative period.

As IRI-induced AKI is commonly encountered in major surgeries such as kidney transplantation, the result of the present study aligns with findings from previous preclinical models of kidney transplantation in which STS protected renal grafts against prolonged cold IRI, leading to improved graft quality, post-transplant outcome, reduced post-transplant complications, and increased survival of transplant recipients [6,21,22,30]. Similarly, a recent experimental investigation also reported the protective effect of STS, which preserved renal mitochondrial integrity against renal IRI in a rat model of a high-fat diet [31]. An interesting observation in the present study is the minimal renal protection provided by one-time dose of STS administration before or after surgical procedure (ischemia and reperfusion), while multiple doses of STS administration (both before and after surgery) produced superior renal protection against IRI-induced AKI. This suggests that whereas renal perfusion and oxygenation remained partially depressed following one-time dose of STS administration before or after surgery, STS administration both before and after the surgical procedure (multiple doses) provided sustained pharmacological support, ensuring continuous renal blood flow, which helped remove toxic products out of the renal tubules, thereby preserving renal structural and functional integrity. Although a one-time dose of STS administration before or after surgery offered some renal protection, we observed no significant difference in renal protection between the two groups. This observation contradicts that of a previous in vitro study in which LLC PK1 cells (epithelial cell line derived pigs) pre-treated with STS protected the cells against IRI while post-treatment did not [32]. The difference in the results of these two studies could be due to differences in experimental settings (in vivo vs. in vitro) and experimental protocols, as STS was administered 30 min before ischemia and 30 min after reperfusion in our in vivo study, while it was administered 60 min prior to ischemia and after reperfusion in the in vitro study. Moreover, the doses of STS used in both studies were different. While there is burgeoning preclinical evidence of STS renal protection against AKI, there is a dearth of evidence supporting its corresponding effect in the clinical literature. So far, only one observational study has reported its renal protective effect in chemotherapy-induced AKI [33].

An important finding in the present study is the upregulation of renal AQP2 gene expression by STS. AQPs are a family of transmembrane proteins that regulate intra- and intercellular water flow, thus influencing the process of urine concentration in the kidneys. Several AQPs are localized at various regions of the nephron. Among them, AQP2 is the major regulator of urine concentration, localized in the principal cells of the collecting duct of the nephron. In a mouse model of lithium-induced nephrogenic diabetes insipidus, a rare water balance disorder characterized by polyuria and polydipsia, Luo and colleagues [34] observed marked upregulation of renal AQP2 protein expression following pharmacological administration of GYY4137, a slow-releasing hydrogen sulfide (H_2_S) donor compound, resulting in significant improvement in urine concentration. Considering that STS is also a slow-releasing H_2_S donor drug, it is not surprising that STS upregulated renal AQP2 gene expression and prevented the development of urine concentration defect in the present study. This was evidenced in the present study by significantly high urine osmolality in STS-treated rats during the 7-day post-operative period compared to untreated control rats, suggesting increased water reabsorption by the collecting duct cells. It further suggests that the observed renal protection and accelerated recovery by STS occurred partly via an H_2_S mechanism. Another important observation in the present study is the high urine production in our IRI-induced AKI control rats compared to STS-treated groups at POD7. Although AKI is associated with reduced glomerular filtration rate (GFR) and oliguria, recovering kidneys can temporarily produce high volumes of urine as they regain function but struggle to properly reabsorb water and electrolytes. This is referred to as the *“diuretic phase”*, where recovery of GFR is faster than the tubules’ reabsorptive function as the kidney heals from AKI [35], hence resulting in “washing out” of excess fluid and accumulated waste. This accounts for the high urine output we observed in the IRI-induced AKI control rats during the post-operative days. Although STS provided renal protection over the 7-day post-operative period, with differences in the degree of recovery from IRI-induced AKI, there are no prior or current data indicating whether the differences we observed in the treatment groups led to any long-term histological changes in the kidney. However, considering the incremental and accelerated improvement in the treatment groups, it is likely that there would be complete histological recovery in these groups if the observation period was extended beyond post-operative day 7.

One of the mechanisms underlying the observed renal protection and accelerated recovery by STS in the present study is preservation of renal antioxidant status throughout the 7-day post-operative period. As our IRI-induced AKI control rats showed elevated levels of reactive oxygen species (ROS; a deleterious mediator of cell and tissue injury) indicated by high levels of malondialdehyde (MDA; lipid peroxidation), renal glutathione (GSH) content and superoxide dismutase (SOD) activity were significantly low in this group of rats. Given that renal mitochondria are a principal contributor to ROS production and oxidative stress in renal IRI, our observation of high ROS levels and low antioxidants in the kidneys of IRI-induced AKI control rats suggests mitochondrial dysfunction and oxidative stress. It further indicates that treatment with STS prevented or neutralized ROS throughout the entire injury and recovery window, thus preserving renal mitochondrial integrity and preventing oxidative stress against development and progression of IRI-induced AKI, and thereby accelerating recovery from IRI-induced AKI. As STS is an H_2_S donor drug, our result is supported by findings from other experimental studies showing that exogenous administration of H_2_S through other H_2_S donor compounds directly scavenged and neutralized ROS and boosted renal antioxidant defenses while at the same time activating the Keap1/Nrf2 pathway, the primary defense mechanism and a major regulator of cytoprotection through activation of several antioxidant enzymes against oxidative stress [36,37,38,39]. Although our study did not explore the Keap1/Nrf2 signaling pathway, it is likely that STS activated this major cytoprotective pathway, as evidenced by increased renal GSH and SOD levels, leading to the observed renal protection against oxidative stress-mediated AKI.

Another important mechanism in the present study is the finding that the observed renal protection and accelerated recovery associated with STS correlated with renal upregulation of PGC1α and SIRT3 gene expression and downregulation of NLRP3 gene expression. SIRT3 (sirtuin 3) and PGC-1α (peroxisome proliferator-activated receptor-gamma coactivator-1 alpha) are important proteins that promote and regulate mitochondrial biogenesis and bioenergetics and inhibit mitochondrial oxidative damage, thereby preserving mitochondrial health and functional integrity under stressful conditions such as in renal IRI-induced AKI. Interestingly, STS upregulated the expression of SIRT3 and PGC-1α genes, which were substantially downregulated in our IRI-induced AKI control rats, consistent with recent experimental models of renal IRI in high-fat diet-fed rats and chemotherapy-induced AKI rats [31,40]. The upregulation of SIRT3 and PGC-1α gene expression by STS in the present study correlated with downregulation of NLRP3 (NLR family pyrin domain containing), a critical complex in the innate immune system that triggers an inflammatory cascade when activated and is involved in the pathogenesis of kidney disease conditions including IRI-induced AKI [41,42]. Hence, we observed significant inflammation in the kidneys of the IRI-induced AKI control rats characterized by huge influx of neutrophils and macrophages (foundational cells of the innate immune system) and release of pro-inflammatory cytokines. As observed in the present study, our result suggests that SIRT3 and PGC-1α are negative regulators of NLRP3, whose activations by STS downregulated NLRP3 gene expression and contributed to the observed protection and accelerated recovery from IRI-induced AKI. In this context, NLRP3 deficiency was recently reported to attenuate renal IRI and enhanced anti-inflammatory response in rats [43].

It is important to note that several signaling pathways regulate oxidative stress and NLRP3 inflammasome activation, as reported in animal models of IRI-induced AKI and other AKI models. For example, the transcription factor nuclear factor-kappa B (NF-kB) acts as a master priming signal that activates the transcription of NLRP3 and increased pro-inflammatory cytokine release in AKI [44,45], while over-production of ROS (oxidative stress) activates mitogen-activated protein kinase (MAPK; a three-tiered protein kinase cascade), which in turn amplifies pro-inflammatory response and triggers mitochondrial-dependent cellular apoptosis in renal tubular cells [46,47]. Also, as a critical regulatory axis in AKI, AMP-activated protein kinase (AMPK; a master cellular energy sensor) protects the kidney and promotes cellular clean-up mechanisms by suppressing NLRP3 activation and NLRP3-driven inflammation and pyroptotic cell death through inhibition of NLRP3 inflammasome expression and assembly in renal tubular cells, while boosting antioxidant defenses and clearing damaged mitochondria by mitophagy and autophagy [48,49]. Under high-oxidative-stress conditions such as in IRI-induced AKI, thioredoxin-interacting protein (TXNIP; a key protein that blocks antioxidant defenses) dissociates from reduced thioredoxin and directly binds to NLRP3, triggering NLRP3 inflammasome assembly and activation, leading to increased release of pro-inflammatory mediators and caspase-dependent cell death and thereby culminating in rapid loss of kidney function during AKI [46,50]. Considering that STS bolstered renal antioxidant status, which correlated with downregulation of NLRP3 gene expression in the present study, it is likely that STS significantly regulated these signaling pathways, thus contributing to the observed kidney protection in the present study.

Despite the promising findings in the present study suggesting potential to protect the kidney and improve post-surgical recovery from IRI-induced AKI, our work has limitations. We could not measure GFR, due to technical challenges. Measurement of GFR would have provided additional information on renal function and the impact of STS on renal hemodynamics. In addition, as STS is an H_2_S donor drug, measurement of renal and plasma H_2_S contents would have provided direct information about H_2_S as one of the major underlying protective mechanisms against IRI-induced AKI. Moreover, the 7-day post-operative period may have been too short to study the complete renal protective effect of STS and fully accelerated recovery from IRI-induced AKI. Future studies should consider extending the post-operative period beyond 7 days. Furthermore, our proposed mechanism of PGC1α/SIRT3/NLRP3 signaling could only be considered as correlational rather than causative, as we did not use knock-out/knock-in animals or gene silencing techniques, which are beyond our technical ability. Given this technical limitation, future studies should consider the use of knock-out/knock-in animals and gene silencing to establish PGC1α/SIRT3/NLRP3 signaling as the causative mechanism of protection. Also, as the present study focused on PGC1α/SIRT3-mediated oxidative stress and NLRP3 inflammasome as the proposed underlying target mechanisms, future studies should explore other signaling pathways underlying the protective effect of STS, which are interrelated with its antioxidant, anti-inflammatory, and anti-apoptotic effects. Moreover, additional functional assays such as ASC oligomerization or speck formation, caspase-1 activation, mature IL-1β and IL-18 secretion, gasdermin D cleavage, and direct evaluation of pyroptotic cell death should be performed in future studies to provide additional evidence that STS inhibits inflammasome activation. Additionally, as the present study relied mainly on conventional oxidative stress biomarkers and antioxidant enzyme activities, future investigations should include direct assessments of mitochondrial function though measurement of mitochondrial membrane potential, ATP production, oxygen consumption rate, mitochondrial morphology, and ultrastructural analysis to substantiate our claim that STS preserves mitochondrial homeostasis in IRI-induced AKI. Finally, although preclinical and clinical studies report that male kidneys are generally more vulnerable to severe IRI and dysfunction [51,52,53], emerging results from a recent clinical study indicate that female kidneys may experience subclinical damage, suggesting that females who suffer AKI may be at a higher risk of progressing to CKD [54]. In the light of this sexual disparity in AKI, further experimental and clinical studies are required to shed more light on AKI-associated sexual dimorphism. Nonetheless, the findings from the present study contribute to the growing body of existing literature on the protective effect and mechanism of STS against IRI-induced AKI.

## 5. Conclusions

The present study provides the first experimental evidence showing that STS administration both before and after a surgical procedure (in multiple doses) provided sustained pharmacological protection and accelerated recovery from IRI-induced AKI. This was evidenced by improved renal structural and functional integrity as well as preserving renal antioxidant status. Based on our experimental data, we identified PGC1α/SIRT3-mediated oxidative stress and downregulation of NLRP3 expression as the potential underlying mechanism, which correlated with the renal protection by STS and accelerated recovery from IRI-induced AKI. However, further studies are needed to establish a causational relationship. Our work demonstrates potential for improved post-surgical recovery, which could be applied in clinical settings with regard to optimum surgical protocols in many clinical conditions involving poor perfusion, such as in kidney transplantation, cardiac surgery, and vascular surgery.

## Figures and Tables

**Table 1:** Primers used for rt-qPCR and accession numbers of analyzed genes.

Sequence	Primer	Accession	Product Size	40 PCR Cycle
CGTGTTCCTACCCCCAATGT	GAPDH F3	NG_149615.1	73	95° for 15 s, 60 °C for 1 min
TGTCATCATACTTGGCAGGTTTCT	GAPDH R3	NG_149615.1	73	95° for 15 s, 60 °C for 1 min
TGGAGTGACATAGAGTGTGCTG	PGC1α-F1	NM_031347.1	239	95° for 15 s, 60 °C for 1 min
GTGAGGACCGCTAGCAAGTT	PGC1α-R1	NM_031347.1	239	95° for 15 s, 60 °C for 1 min
TGGGGTCCGGGAGTATTACA	SIRT3-F1	NM_001106313.2	82	95° for 15 s, 60 °C for 1 min
TGTCCTCCACATCCAAAGCC	SIRT3-R1	NM_001106313.2	82	95° for 15 s, 60 °C for 1 min
ACCCGAGAAAGGCTGTGTTC	NLRP3-F1	XM_063268608.1	201	95° for 15 s, 60 °C for 1 min
GACGTGCATGCATCATTCCA	NLRP3-R1	XM_063268608.1	201	95° for 15 s, 60 °C for 1 min
AGCAGCATGTGGGAACTCAG	AQP2 F2	NM_012909.3	380	95° for 15 s, 60 °C for 1 min
GCGTTGTTGTGGAGAGCATTG	AQP2 R2	NM_012909.3	380	95° for 15 s, 60 °C for 1 min
CGGCTAACCAGAGTGACTTGT	KIM-1-F2	NM_173149.2	100	95° for 15 s, 60 °C for 1 min
TACAGAGCCTGGAAGAAGCAG	KIM1-R2	NM_173149.2	100	95° for 15 s, 60 °C for 1 min
GGGCTGTCCGATGAACTGAA	NGAL-F2	NM_130741.2	98	95° for 15 s, 60 °C for 1 min
CATTGGTCGGTGGGAACAGA	NGAL-R2	NM_130741.2	98	95° for 15 s, 60 °C for 1 min
TGGTCTTCTGGAGTTCCGTT	IL-6-F3	NM_012589.2	216	95° for 15 s, 60 °C for 1 min
GTCCTTAGCCACTCCTTCTG	IL-6-R3	NM_012589.2	216	95° for 15 s, 60 °C for 1 min

## Data Availability

The original contributions presented in this study are included in the article. Further inquiries can be directed to the corresponding author.
